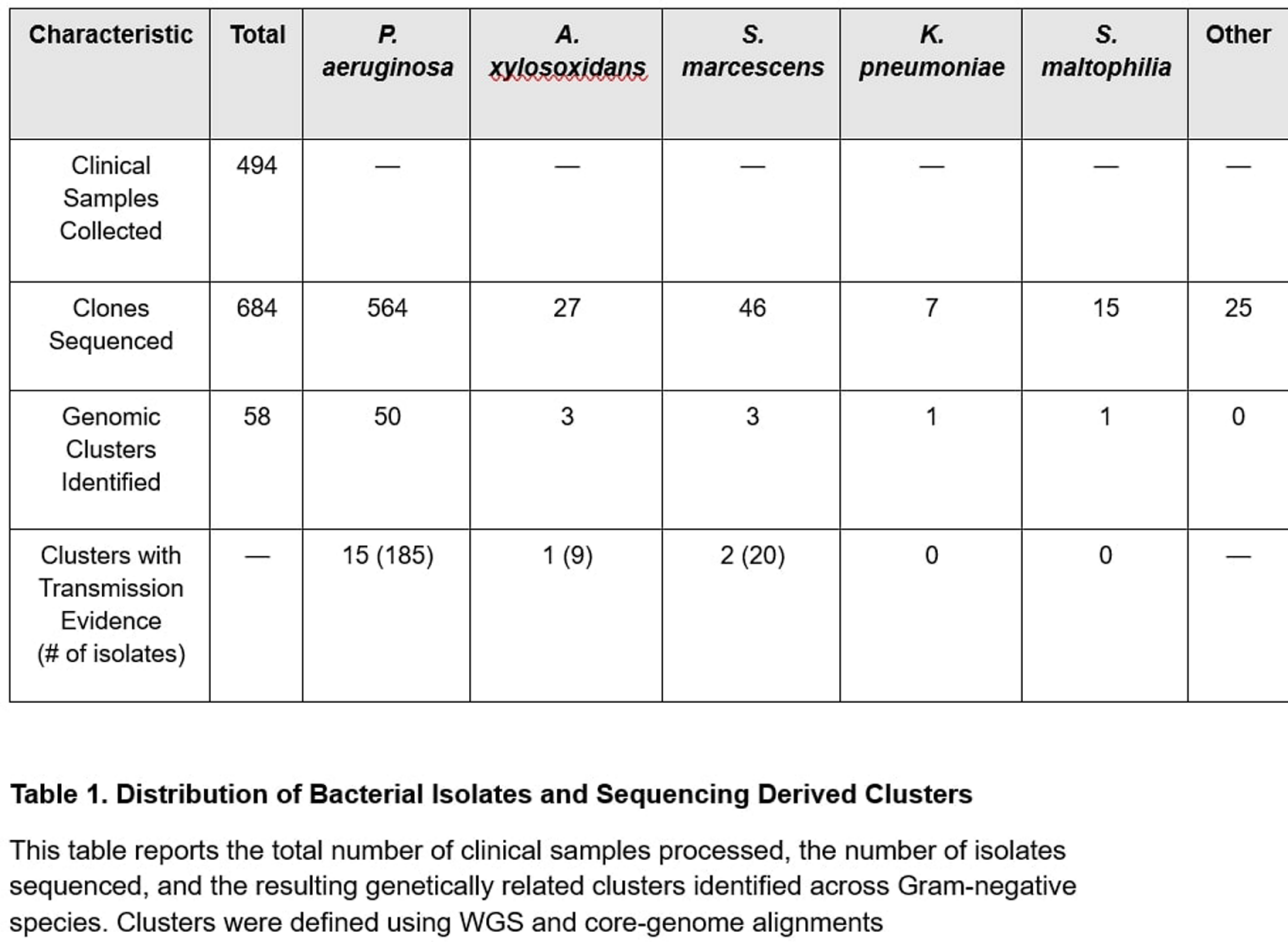# 230 Utilization and Yield of a Stool Enteric Pathogen Panel in Hospitalized Patients with Cancer with Community vs Hospital-onset Diarrhea

**DOI:** 10.1017/ash.2026.10729

**Published:** 2026-06-23

**Authors:** Jacob Scioli, Erin Nawrocki, Luke Kiesendahl, Lindsay Montoya, Vaughn Cooper, Glenn Rapsinski

**Affiliations:** 1 University of Pittsburgh School of Public Health; 2 University of Pittsburgh; 3 University of Pittsburgh School of Health and Rehabilitation Sciences; 4 UPMC Children’s Hospital of Pittsburgh; 5 University of Pittsburgh School of Medicine

## Abstract

**Background:** Hospital-acquired infections remain a significant challenge for healthcare systems despite well-established infection prevention (IP) strategies, with ventilator-associated infections (VAI) being particularly challenging. This is especially true for pediatric patients, who often require mechanical ventilation for extended periods, increasing the risk of infection. Gram-negative bacteria, primarily Pseudomonas aeruginosa, are the leading causes of VAI. Traditional IP strategies are often reactive and may miss potential transmissions within the hospital, particularly for pathogens that are endemic to the hospital environment. Whole-genome sequencing (WGS) and genomic epidemiology have demonstrated value in identifying such events in adult hospitals, but their application in pediatric facilities remains limited. Here, we conduct a retrospective analysis to determine how this technology could be utilized in a pediatric facility. **Methods:** We conducted a retrospective genomic surveillance study of Gram-negative isolates collected between 2020 and 2024 at a pediatric tertiary hospital. Briefly, clones were selected by colony morphology, cultured, and DNA was extracted. DNA libraries were prepared and sequenced on the Illumina NextSeq 1000 platform. Phylogenetic analyses were performed using core-genome alignment pipelines. Likely transmission events were defined as bacterial clones differing by fewer than 10 single-nucleotide polymorphisms, based on previously described literature. Genetically related clones were evaluated with patient location and temporal hospital metadata to assess epidemiologic linkage. **Results:** A total of 687 clones from roughly 500 clinical samples were sequenced. We identified over 50 groups of genetically related isolates from multiple Gram-negative species. P. aeruginosa accounted for most of the genetically related groups, with over 40 discovered. Additional pathogens suspected to have genetic relatedness include Serratia marcescens, Klebsiella pneumoniae, Stenotrophomonas maltophilia, and Achromobacter xylosoxidans. These groups range in size from 2 to 15 patients. Pairwise genomic analysis confirmed at least 16 groups consistent with likely transmission events. To date, epidemiologic review has confirmed that 2 of these groups were plausible based on temporal and geographic relationships between patients. The remaining 14 groups are under investigation. **Conclusion:** WGS-based surveillance identified multiple transmission events that were not recognized by conventional IP methods, highlighting the added value of WGS for hospital surveillance. This is especially true in the case of pathogens that are commonly found in hospitals. Integrating WGS with genomic epidemiology and hospital metadata can enhance patient safety and reduce healthcare costs. These findings indicate that reliance on traditional surveillance alone may underestimate the true burden of hospital outbreaks, particularly those caused by common Gram-negative respiratory pathogens in pediatric populations.

**Figure:**